# Whole Brain Cellular Activation After Mu and NOP Receptor Agonism Identifies Differential Regional and Network Consequences

**DOI:** 10.64898/2026.09.22.753526

**Published:** 2026-09-28

**Authors:** Madeline Martinez, Akihiko Ozawa, Daniel Van Zant, Jake Thornberry, Lawrence Toll

**Affiliations:** 1 Department of Biomedical Science, Schmidt College of Medicine, Florida Atlantic University, Boca Raton, FL 33431, USA; 2 Stiles-Nicholson Brain Institute, Florida Atlantic University, Jupiter, FL 33458, USA

## Abstract

Mu opioid agonists, the most widely used opioids, produce analgesia, respiratory depression, constipation, and reward leading to substance abuse. Like all opioid receptor family members, mu receptors are Gi/o-coupled and inhibitory, yet mu-mediated disinhibition of GABAergic neurons propagates downstream activation that differs between naive and dependent animals and across receptor subtypes. Here we used TRAP2/Ai9 reporter mice, which deposit tdTomato in active neurons via the c-Fos locus, with tissue clearing and light sheet microscopy to map whole-brain neuronal activation after acute morphine (10 mg/kg), an escalating twice-daily morphine regimen producing dependence, or the NOP receptor agonist Ro 64–6198. Despite Gi/o coupling, all treatments raised global activation relative to vehicle, though this whole-brain increase reached significance only in males, and acute morphine engaged the largest number of regions, all increased with none decreased. Sex differences were prominent: males were more sensitive than females across treatments, with morphine engaging mainly pain- and reward-related midbrain circuitry in males and hypothalamic circuitry in females. In dependent mice, activation was lower across several regions, including a cluster of cerebellar regions that trended toward being turned off relative to acute morphine. NOP receptor activation produced a far weaker profile. Although chronic morphine produced less activation than acute morphine, it produced more widespread co-activation, and while hub composition differed across conditions, global network organization was preserved. These results reveal whole-brain changes in neuronal activation and establish a TRAP2/Ai9 model that renders activated regions genetically accessible for future site-directed investigation.

## Introduction

Opioid receptors are found throughout the brain and spinal cord, as well as in the periphery, consistent with the wide range of biological and physiological processes opioids influence, including pain, motivation, stress, respiration, and gastrointestinal mobility [1–3]. The three classical opioid receptors—mu, kappa, and delta—together with the nociceptin/orphanin FQ peptide (NOP) receptor make up the opioid receptor family, a group of Gᵢ/₀-coupled receptors activated by known endogenous peptides [4–6]; yet nearly all clinically used medications are mu agonists. Morphine and analogs, through activation of mu receptors, are the most successful compounds for moderate-to-severe pain, but activation of these same receptors leads to serious complications including constipation, respiratory depression, reward and addiction [7]. Agonists and antagonists of other receptors in the family have been developed for treatment of pain and other disorders but have been generally unsuccessful. Recently, the NOP receptor has drawn interest as a target for analgesia and substance-use medications, because NOP activation modulates reward processing and can temper the abuse liability and side-effects of mu agonists [8–9]. As bifunctional NOP/mu agonists such as cebranopadol advance toward clinical use, a clearer understanding of how NOP receptor activation reshapes activity across the brain becomes increasingly valuable [10–11].

One limitation in understanding effects of acute and chronic opioid treatment has been inability to quantify cellular activation across the whole brain. This matters given the wide distribution of opioid receptors, whose activation alters activity at cellular, regional, and network levels. Opioid effects at the network level have been examined using fMRI in both humans and rodents, providing considerable information about resting state and drug-evoked functional connectivity among brain regions [12–13]; however, fMRI lacks the resolution to investigate changes in activation at the cellular level. At cellular resolution, the primary approach has been to visualize the immediate early gene product Fos as a proxy for recent neuronal activity, including at the whole-brain level [14]. More recently, mouse genetic models, such as TRAP (Targeted Recombination of Active Populations), in which CreER recombinase is expressed at the c-fos locus, and newer versions such as TRAP2/Ai9 mice, in which tamoxifen drives permanent tdTomato expression in neurons active during a defined window, have been used extensively [15–19]. This approach allows for a permanent label during a specific time window, but also further genetic manipulation due to the presence of Cre deposited in the activated neurons. The same strategy has been used to identify neurons activated by a discrete stimulus and then manipulate them with DREADDs [18] or optogenetics [20].

Recent advances in optical clearing, light sheet microscopy, and automated cell counting have made it feasible to quantify tdTomato-labeled neurons throughout the brain. Because TRAP2/Ai9 yields a bright, endogenous tdTomato signal, cleared tissue can be imaged directly, without immunolabeling on which whole-brain Fos mapping has relied [21–23], thus removing any dependence on antibody selectivity or penetration into deep and internal structures. In this manuscript we use light sheet microscopy following clearing with a modified version of iDISCO adapted to preserve native fluorescence (MDISCO) [24] to quantify neuronal activation throughout the mouse brain after treatment with acute morphine, escalating morphine doses to induce dependence, the selective NOP agonist Ro 64–6198, and vehicle. When registered to the Allen Common Coordinate Framework (CCFv3) [25], sex-dependent changes in individual brain regions and functional co-activation were identified, with altered numbers of activated cells after drug treatment, while global network topology and modular organization were largely preserved, consistent with known behavioral, physiological, and neuroimaging observations in humans.

## Materials and Methods

All animal procedures were approved by Florida Atlantic University IACUC and conducted in accordance with NIH guidelines. Male and female TRAP2 (Fos2A-iCreER) × Ai9 (Rosa26-LSL-tdTomato) mice (n = 6 per sex per condition, except Morphine-Dependent, n = 5) were assigned to four conditions: Vehicle, Acute Morphine (10 mg/kg, i.p.), Morphine-Dependent (an escalating twice-daily morphine regimen, 10 to 75 mg/kg across Days 1–4 followed by a single 25 mg/kg injection on Day 5), or the nociceptin/orphanin FQ (NOP) receptor agonist Ro 64–6198 (0.6 mg/kg, i.p.) [26]. The escalating-dose regimen represents an established method to induce opioid physical dependence in rodents [27]; the reduced group size for this condition (n = 5 per sex) reflects loss of animals during the high-dose phase. Neurons active during drug exposure were labeled genetically rather than by immunostaining: 4-hydroxytamoxifen (50 mg/kg, i.p.), administered 30 min after the drug, drove permanent c-Fos–dependent tdTomato expression, and mice were perfused 10 days later. Whole brains were cleared with MDISCO [24], imaged intact on a Miltenyi UltraMicroscope Blaze II light-sheet microscope and processed in NeuroInfo [28] for automated detection of tdTomato+ somata and registration to the Allen CCFv3 [25]. Automated detection and atlas-registration pipeline have been validated against manual delineation and reference-atlas landmarks [28–29], with localization accuracy lower for small ventrolateral and midline structures. The complete experimental and analytical workflow is summarized in Figure 1. Counts were restricted to grey-matter regions and averaged across hemispheres; bilateral sums were used for the negative binomial model, which requires integer counts. Regions were then filtered to remove the ventral-most structures obstructed at the imaging mounting surface and required to have bilateral representation in every export (16 further regions were absent or unilateral and were therefore not analyzed), yielding 198 regions for analysis (Table S1). Regions were analyzed at a single, consistent level of the CCFv3 summary-structure hierarchy, so that no analyzed region is nested within another and interregional co-activation is not inflated by anatomical containment.

Regional activity was analyzed as c-Fos+ density (cells/mm^3^, log_10_-transformed). The primary test was a one-way ANOVA across conditions (sexes pooled) with Tukey contrasts and Benjamini-Hochberg FDR across regions (q < 0.05); sex-stratified and condition × sex two-way ANOVAs were secondary and supplemental analyses, and a negative binomial GLM on bilateral summed counts, with log(region volume) as an offset, confirmed that primary findings were not an artifact of the log_10_ transformation. Interregional co-activation networks were constructed from across-animal Pearson correlations (combined, sex-residualized network as primary; per-sex networks reported as supplemental confirmation) and matched to a common 10% edge density. Networks were modular relative to degree-preserving nulls and small-world; condition differences in co-activation were tested edge-wise by Fisher r-to-z with FDR correction, and differences in global network metrics by bootstrap confidence intervals and label-permutation tests (strength and topology corrected as separate families). Detailed procedures for clearing, imaging, registration, normalization, network construction, node-role assignment, and statistics are provided in Supplementary Methods.

Generative AI was used during manuscript preparation and in preparing the analysis code for public deposit. A detailed description of AI use can be found in Supplementary Information.

## Results

### Acute morphine engages a hypothalamic–preoptic–brainstem network with sex-specific regional emphasis

When sexes were combined, acute morphine produced a non-significant 1.37-fold increase in whole-brain total tdTomato^+^ (c-Fos-expressing) cells relative to vehicle (one-way ANOVA F(3,42) = 2.41, p = 0.081; Figure S1C). This and other pooled results obscured a substantial difference in the magnitude of the response between the sexes. The pooled regional results are presented in Supplementary Information (Figure S1A,D).

#### Sex-specific analysis.

Morphine-induced activation was in the same direction in both sexes but differed in breadth and magnitude, with males showing considerably greater tdTomato expression than females. This asymmetry is apparent in the whole-brain total: the number of morphine-activated cells rose significantly in males (1.66-fold; one-way ANOVA F(3,19) = 3.58, p = 0.033; Tukey Vehicle vs. Morphine p = 0.019) but not in females (1.15-fold; F(3,19) = 0.34, p = 0.80) (Figure 2B), so the non-significant pooled total (Figure S1C) reflects a real male increase diluted by a flat female response. In within-sex ANOVAs, males engaged a broad set of 82 regions (20 FDR-significant despite n=6 per group rather than n=12 in the pooled analysis), with the most highly activated regions encompassing midbrain structures; collicular, septal, and a distinctive hippocampal-subicular cluster (presubiculum, postsubiculum, dentate gyrus, CA fields) were not detected in females, together with basal ganglia, VTA, and descending pain circuitry (PAG, PPN, RN) (Figure 2A). Top male effects exceeded |d| = 2.5, exemplified by the substantia nigra reticular part and presubiculum (SNr and PRE; Figure 2C; Table S2). Females reached significance (Tukey-adjusted p < 0.05, not corrected across regions) in only 9 regions, none surviving FDR, concentrated in preoptic and periventricular hypothalamic targets; the anteroventral periventricular nucleus showed the largest female effect (AVPV; |d| = 2.22, well above its combined-sex estimate, Figure 2C). Two-way ANOVA revealed no condition × sex interaction surviving FDR at any region (q > 0.71; 12 nominal at uncorrected p < 0.05).

### Morphine dependence sustains the hypothalamic–preoptic activation core with sex-divergent network footprints

Dependent animals also showed sex differences in regional neuronal activation. Males showed 38 regions at Tukey p < 0.05 (none FDR-significant), with the largest effects extending beyond the core into septal, collicular, and midbrain nodes (triangular nucleus of septum |d| = 3.74) while the core itself remained strongly represented (Table S2). Females showed only 7 regions (none FDR-significant), again concentrated at preoptic-hypothalamic targets — the same narrow, large-effect footprint seen acutely. Direct comparison of the two morphine conditions revealed a non-significant decrease in total activation and no region surviving FDR whether looking at pooled or individual sex data (r = 0.35 across all 198 regions, 0/198 FDR-significant), although a set of cerebellar, pontine, and medullary structures showed lower c-Fos^+^ density in dependent animals compared to acute morphine or vehicle treatment (Tukey p < 0.05), an effect driven entirely by males and exemplified by the paramedian lobule (PRM; Figure 2C).

### NOP receptor agonism produces a pharmacologically distinct activation profile

Acute Ro 64–6198 (0.6 mg/kg, analgesic but not sedative [26]) reached significance (Tukey p < 0.05) in only 3 regions versus vehicle (MBO [Figure 2D], diagonal band nucleus, intertrigeminal nucleus), none surviving FDR — compared to 84 under acute morphine and 49 under dependence at the same threshold (Figure S1B; Table S2). Effect sizes nonetheless remained positive in 99.5% of regions but smaller than morphine’s (mean |d| 0.56 vs 0.98) — a weaker, not anatomically restricted, response. Sex-stratified analysis again showed a marked dissociation: males engaged 18 regions (none FDR-significant) overlapping the male brainstem-mesencephalic morphine signature (SAG, SNr, PRE, SCs, all |d| > 2.2), whereas females showed no region at Tukey p < 0.05. AVPV, the largest female node under acute morphine, showed a comparable female effect size under Ro 64–6198 (|d| = 1.56) but did not reach significance.

### Widespread increases in co-activation under morphine dependence occur without changes in global network organization

The regional and network analyses diverged informatively: acute morphine produced the broadest change in individual-region activity, whereas dependence produced, by far, the largest change in inter-regional co-activation despite engaging fewer regions. To ask how drug treatment reorganizes coordination between regions, we built co-activation networks from across-animal correlations in regional activated-cell counts (Pearson r; region pairs varying in tandem treated as ‘co-activated’), pooling all animals after mean-centering each region within sex to remove overall male–female differences. Per-sex networks confirmed that combined-network results were maintained within each sex (Supplementary Methods). In the combined-sex data, mean absolute correlation across all 19,503 region pairs was lowest under vehicle (|r| = 0.45), intermediate after acute morphine (0.52), far higher in dependent animals (0.74), and near vehicle levels after Ro 64–6198 (0.50; Figure 3A,B). For topological comparisons, networks were thresholded to the strongest 10% of links (matched density [30]). This rise in mean correlation was broad-based rather than system-specific, with within-division co-activation increasing in dependent animals in 11 of the 12 divisions shown, isocortex being the exception (Figure 3D). The increase in co-activation in dependent animals was present in both sexes, and numerically larger in females (Figure 3B; per-sex matrices, Figure S2).

Edgewise comparison (Fisher r-to-z, BH-corrected) localized these differences in co-activation strength almost entirely to dependence, as the vehicle versus morphine-dependent comparison yielded, in the combined-sex network, 160 region pairs with significantly altered co-activation, every one of them stronger in dependent animals. Every other pairwise comparison among the four conditions (e.g., vehicle vs. acute morphine, vehicle vs. Ro 64–6198, acute vs. dependent) yielded ≤ 3 such pairs (Figure 3C; Table S3). Because correlation strength is sensitive to sample size and the dependent group had the smallest n, magnitudes are interpreted descriptively; the robust observation is that co-activation increases, not decreases, in dependent animals.

### Global network organization is preserved while hub composition differs across conditions

Although dependence sharply increased co-activation strength, we detected no accompanying change in the network’s overall architecture — how it organizes into communities or how efficiently it is wired. In all four conditions, the networks shared two features typical of brain networks. They were modular — regions clustered into communities far more than in degree-matched random networks (z = 54.9–78.9 combined; z = 51.2–82.6 per sex; all p < 0.001) — and small-world, combining dense local clustering with short paths between distant regions (σ > 1 in all networks; Table S4). Summary measures of architecture did not differ across conditions: node strength, transitivity, global efficiency, and modularity Q were all statistically indistinguishable across treatments (permutation tests, all q ≥ 0.185; overlapping bootstrap 95% CIs).

A community-detection algorithm (consensus Louvain) split each combined-sex network into 5–8 modules (5–10 across the per-sex networks) that were stable across treatments. The module assignment of each region under each condition is reported in Table S5. We summarized each region’s position in the network with two standard cartographic measures [31]: the participation coefficient (PC), which is high when a region’s connections are distributed across multiple modules (a between-module ‘connector’), and the within-module degree z-score (WMDz), which is high when a region is strongly connected within its own module (a ‘provincial hub’). Their anatomical distribution across conditions is mapped for the combined network (Figure 4A,B) and for each sex, where the male and female maps diverge substantially (Figure S3). The per-sex maps diverged most in isocortex, where acute morphine produced numerous high-PC connector regions in females but few in males, though the reduced per-sex sample precludes formal comparison. The joint PC–WMDz distribution used to classify cartographic roles is shown in Figure S4. The vehicle network was dominated by locally connected regions with few bridges (Figure 4C), with bridging ‘connector’ regions (high PC) becoming more common after acute morphine treatment (median PC 0.20 vs. 0.42; Figure 4A); Ro 64–6198 treatment induced regions that were highly connected but mainly within their own module (high WMDz; ‘provincial hubs’). Hub composition also differed. By the multi-centrality convergence criterion (agreement across four centrality measures; Supplementary Methods), anterior cingulate and prelimbic cortex were hubs in all four conditions, while several cortical, olfactory, and hippocampal hubs of the drug-naive network dropped out in dependent animals. In Figure 4C, rings mark all hub-role regions (WMDz ≥ 1.5) and labeled nodes are the connector-hub subset. Connector hubs under acute morphine were hypothalamic (lateral hypothalamic area, perifornical nucleus) together with midbrain and pontomedullary nodes; under dependence they were hypothalamic (dorsomedial and anteroventral periventricular nuclei), hippocampal, striatal (nucleus accumbens), and medullary. Per-sex networks confirmed this pattern but with less stable hub assignments (no region was a hub in all four conditions in males, and only anterior cingulate in females); female multi-centrality hub counts rose under each drug condition (24 under vehicle vs. 31–35 under morphine and Ro 64–6198) relative to the more stable male count (32–37).

## Discussion

Like many GPCRs, mu opioid receptors are distributed throughout the brain, and like the other members of the opioid receptor family (kappa, delta, and NOP), they are Gi/o coupled, so that receptor activation lowers cAMP, increases GIRK (G protein coupled, inwardly rectifying potassium) channel conductance, and generally inactivates the cell [32]. Despite this inhibitory coupling, morphine-mediated disinhibition of GABAergic neurons raises extracellular dopamine in the nucleus accumbens, producing reward and abuse liability [33–36], and when injected into the rostral ventromedial medulla (RVM) activates “off” cells in the descending pain modulation pathway producing analgesia [37–39]. Therefore, mapping where and how strongly neurons are activated is central to understanding opioid action. Such activation has most often been inferred from the immediate-early gene c-Fos, measured by in situ hybridization or immunohistochemistry [40–43]. Although these techniques visualize neuronal activation, temporal uncertainties and antibody variability complicate interpretation. The recent utilization of TRAP2 and TRAP2/Ai9 mice addresses both limitations, providing a permanent, genetically encoded record of neurons active within a defined window after a known stimulus, together with genetic access to those neurons for subsequent manipulation [16–18].

Combined with advances in tissue clearing and light sheet imaging, the use of TRAP2/Ai9 mice has enabled comprehensive whole-brain analysis of state-dependent cellular and regional neuronal activation, neuronal ensembles and interregional co-activation [44]. fMRI has identified connectivity changes associated with opioid treatment and opioid use disorder in humans [12–13] but lacks the cellular resolution and experimental control achievable in mice.

Here, whole-brain activation was mapped in TRAP2/Ai9 mice across four conditions: vehicle, acute morphine, an escalated regimen producing dependence, and acute treatment with the NOP receptor agonist Ro 64–6198. One major finding was that both morphine and Ro 64–6198 induce an increase in the global number of activated neurons, although this increase in whole-brain activation reached significance only in males, with morphine being more effective than Ro 64–6198. This is consistent with NOP receptor agonist activity being more modulatory [45], with receptors on both GABAergic (to disinhibit) and dopamine neurons (to inhibit) in the VTA [46] and on both analgesic “primary cells” and nociceptive “secondary cells” in the PAG [47], while mu receptors act on GABAergic interneurons within these circuits, contributing to disinhibition-mediated analgesia and reward [33, 47]. These studies also suggest that chronic treatment, in the absence of withdrawal, reduces the level of neuronal activation compared to a single morphine administration, in keeping with the microarray studies [48], likely due to tolerance development. Because activation was captured within a single TRAP labeling window, these maps represent one temporal snapshot of drug action. This window, approximately 6 h initiated by the 4-OHT injection [16], is considerably broader than the 90–120 min post-stimulus interval typical of c-Fos immunostaining.

A second major finding was a pronounced sex difference in the response to 10 mg/kg morphine. Acute morphine significantly increased activation in many more brain regions in males than in females. This difference in breadth parallels the difference in global activation, which rose significantly in males but not in females (Figure 2B). Although the pooled analysis identified hypothalamic regions, extensive midbrain, collicular, and hippocampal–subicular circuitry predominated in males, consistent with higher mu-opioid receptor density in the PAG of male rats [49] and greater intracerebroventricular analgesic potency in male C57BL/6 mice [50]. However, such sex differences in morphine analgesia are strain- and species-dependent, with no sex differences in receptor expression or systemic morphine-induced analgesia in this strain [51]. Female cellular activation was concentrated in preoptic and periventricular hypothalamic targets, with no increase in midbrain activity. Morphine strongly engages hypothalamic neuroendocrine circuits that are modulated by sex hormones, with estrogen modulating mu-opioid receptor signaling in these circuits [52]. Notably, no region showed a condition × sex interaction surviving correction, indicating that both sexes engaged the same network in the same direction, differing in the breadth and magnitude of recruitment rather than in opposing responses.

A third major finding emerged only when activity was examined across regions rather than within them. Whereas acute morphine produced the broadest change in the number of activated regions, it was morphine dependence that produced the largest change in the coordination of activity between regions. In dependent animals of both sexes, the average correlation in activation between region pairs rose well above vehicle, acute morphine, and Ro 64–6198 levels. Dependence thus produced a more coordinated, less variable pattern of activation across animals. This separation suggests that dependence reorganizes the relationships among regions more than it changes which regions are recruited.

A rise in interregional co-activation with dependence is consistent with whole-brain studies of alcohol, in which dependence and abstinence likewise increase cross-correlation between regions [21–22, 53]. In those studies, the increase in coupling was accompanied by an apparent reorganization of network architecture, most often a collapse of modular structure. We did not observe such a change, and the difference is most likely methodological. In the previous studies, networks were built at a fixed correlation threshold, so a condition with higher overall correlation necessarily produces a denser graph, and density strongly shapes measures of modularity and efficiency. We held density constant across conditions, so that architecture could be compared independently of correlation strength. Under these conditions, the coarse organization of the network did not change. Across all four conditions the networks remained modular and small-world, and summary measures of how efficiently activity could spread, how strongly regions clustered, and how cleanly the network divided into communities were statistically indistinguishable.

What did change was the identity of the most highly connected hub regions. Anterior cingulate and prelimbic cortex served as hubs in every condition, but under morphine several cortical, olfactory, and hippocampal hubs of the drug-naive brain gave way to hypothalamic and amygdalar regions — the same territories that dominated the regional analysis. The network and regional results therefore converge on a common set of hypothalamic and amygdalar structures, with prominent midbrain engagement in males, as central to the morphine-activated brain. Notably, these were not the regions most often associated with opioid reward or dependence: the nucleus accumbens and ventral tegmental area, and the central amygdala and bed nucleus of the stria terminalis, all shifted in the same direction as the surviving regions but did not themselves reach significance after correction in the pooled analysis. In males alone, the nucleus accumbens, VTA, and bed nucleus of the stria terminalis were nominally significant (Cohen’s d = 1.6–1.8) but were essentially absent in females (d < 0.4). What survived correction, given the limited sample, was a predominantly neuroendocrine and hypothalamic-integrative profile, comprising preoptic and hypothalamic nuclei together with the medial and basomedial amygdala and adjacent basal forebrain. Because correlation-based measures are sensitive to sample size, we interpret the magnitude of these effects descriptively; the robust observation is that dependence increases the coordination of brain-wide activity without dismantling its global architecture.

Several hub regions, notably the lateral hypothalamus and amygdala, also feature among the durable morphine-associated networks from resting-state fMRI in a mouse model of morphine abstinence [13], though those signatures center on the retrosplenial cortex and default mode network rather than hypothalamic circuitry. Because co-activation quantified across animals is related but not identical to within-subject functional connectivity these fMRI studies report, and because such small, deep nuclei are difficult to resolve with human imaging, whole-brain cellular mapping and fMRI are best regarded as complementary levels of description. Consistent with its weaker regional activation, Ro 64–6198 left interregional co-activation close to vehicle levels, reinforcing that NOP activation engages the brain in a more modulatory fashion than morphine.

Taken together, these results show that morphine, and to a lesser extent NOP receptor activation, reshapes brain-wide activity at multiple levels — the number and identity of activated regions, the strength of their coordination, and the composition of network hubs — in a drug- and sex-dependent manner — with the canonical reward circuitry (NAc, VTA, BNST) and midbrain engaged in males but largely absent in females at this dose of morphine. The convergence of the regional and network analyses on hypothalamic and amygdalar circuitry, and the emergence of heightened interregional co-activation as the dominant signature of dependence, mark these features as priorities for mechanistic follow-up. More broadly, this work establishes whole-brain, cellular-resolution activation mapping as a framework for dissecting opioid action across the entire brain. Set against the weaker, modulatory signature of NOP receptor activation, these results begin to define how distinct members of the opioid receptor family reshape the brain, a distinction that will grow in importance as bifunctional NOP/mu compounds advance toward the clinic [54–55]. Because the TRAP2/Ai9 strategy permanently tags the neurons engaged under each condition, these populations are now genetically accessible for causal, circuit-level dissection, and maps presented here provide a whole-brain, cellular-resolution foundation for resolving how opioids and NOP ligands remodel brain activity across analgesia, tolerance, and dependence.

## Supplementary Material

Supplement 1

Supplement 2

Supplement 3

Supplement 4

Supplement 5

Supplement 6

Supplementary Tables S1–S5, Supplementary Figures S1–S4, and the Supplementary Methods are provided in the Supplementary Information.

## Figures and Tables

**Figure 1.**
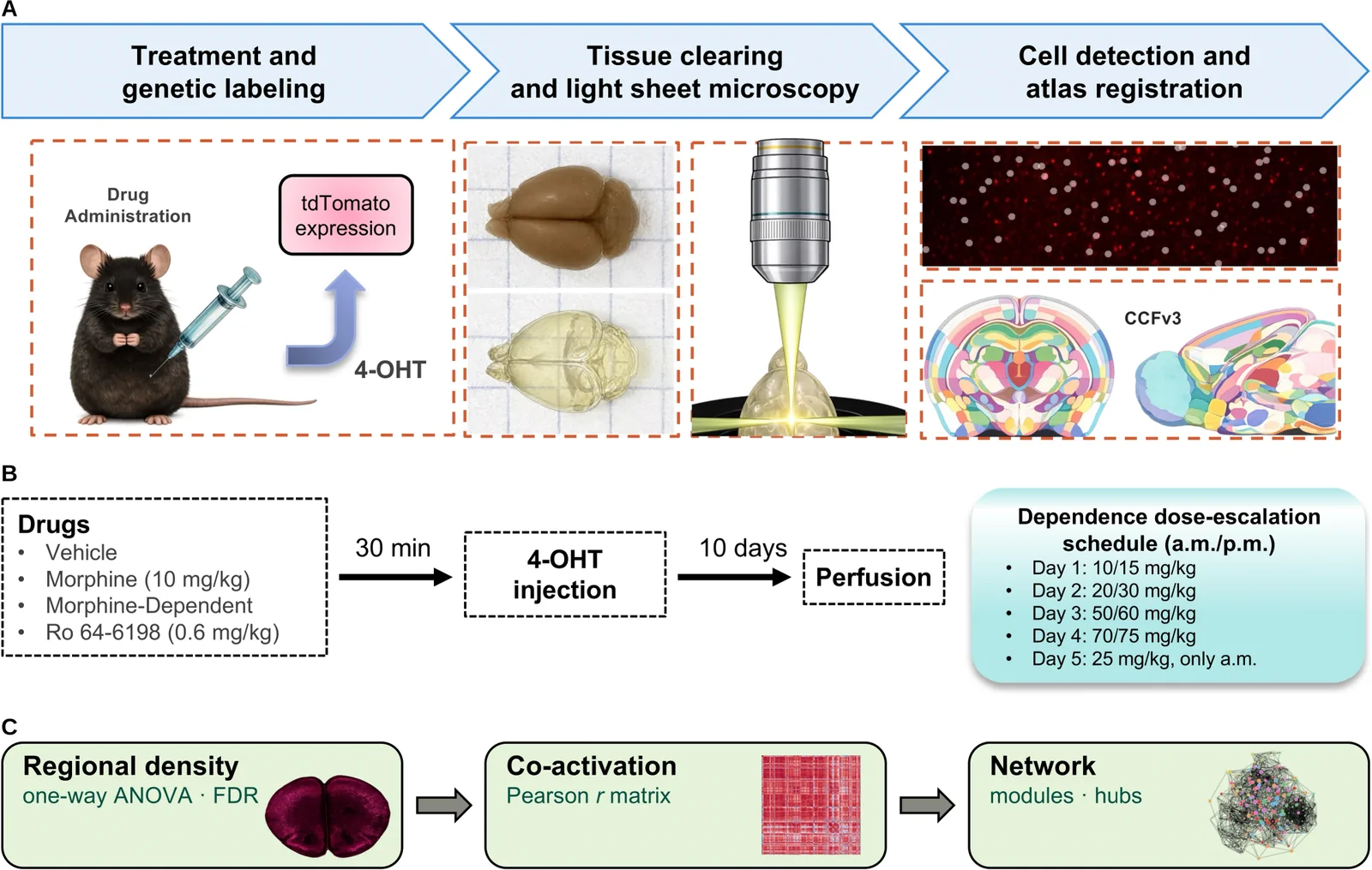
Experimental design and analysis workflow. (A) Whole-brain cellular activity mapping pipeline. TRAP2/Ai9 mice (Fos2A-iCreER × Rosa26-LSL-tdTomato) received drug treatment followed by 4-hydroxytamoxifen (4-OHT; 50 mg/kg, i.p.), permanently labeling neurons active during the labeling window with tdTomato. Brains were cleared with MDISCO, a modified iDISCO protocol adapted to preserve native fluorescence, and imaged intact by light-sheet fluorescence microscopy (Miltenyi UltraMicroscope Blaze II). tdTomato-positive cells were detected and registered to the Allen Mouse Brain Common Coordinate Framework (CCFv3), yielding cell counts and densities across 198 grey-matter regions. Left, drug administration and 4-OHT-dependent tdTomato labeling. Center, uncleared (top) and cleared (bottom) brain. Right, illustrative example of tdTomato signal with NeuroInfo-detected somata overlaid (grey; scale bar, 100 μm), and the CCFv3 coronal and sagittal reference atlas. (B) Treatment timeline. Mice received vehicle, acute morphine (10 mg/kg, i.p.), morphine on an escalating dependence-inducing regimen, or the selective nociceptin/orphanin FQ peptide (NOP) receptor agonist Ro 64–6198 (0.6 mg/kg, i.p.), followed 30 min later by 4-OHT to open the labeling window. Brains were collected 10 d later. Right, twice-daily (a.m./p.m.) dose-escalation schedule for the morphine-dependent group, culminating in a single 25 mg/kg challenge on the morning of Day 5. Group sizes: Veh, Mor, and Ro, n = 12 (6 per sex); MorDep, n = 10 (5 per sex). (C) Analysis pipeline. Regional tdTomato-positive cell density was compared across conditions by one-way ANOVA on log_10_-transformed density, with Benjamini-Hochberg FDR correction applied across all 198 regions. Interregional co-activation was quantified as the Pearson correlation (r) between regional densities across animals within each condition. Correlation matrices were thresholded at 10% matched density and analyzed as graphs to characterize modular organization and hub structure.

**Figure 2.**
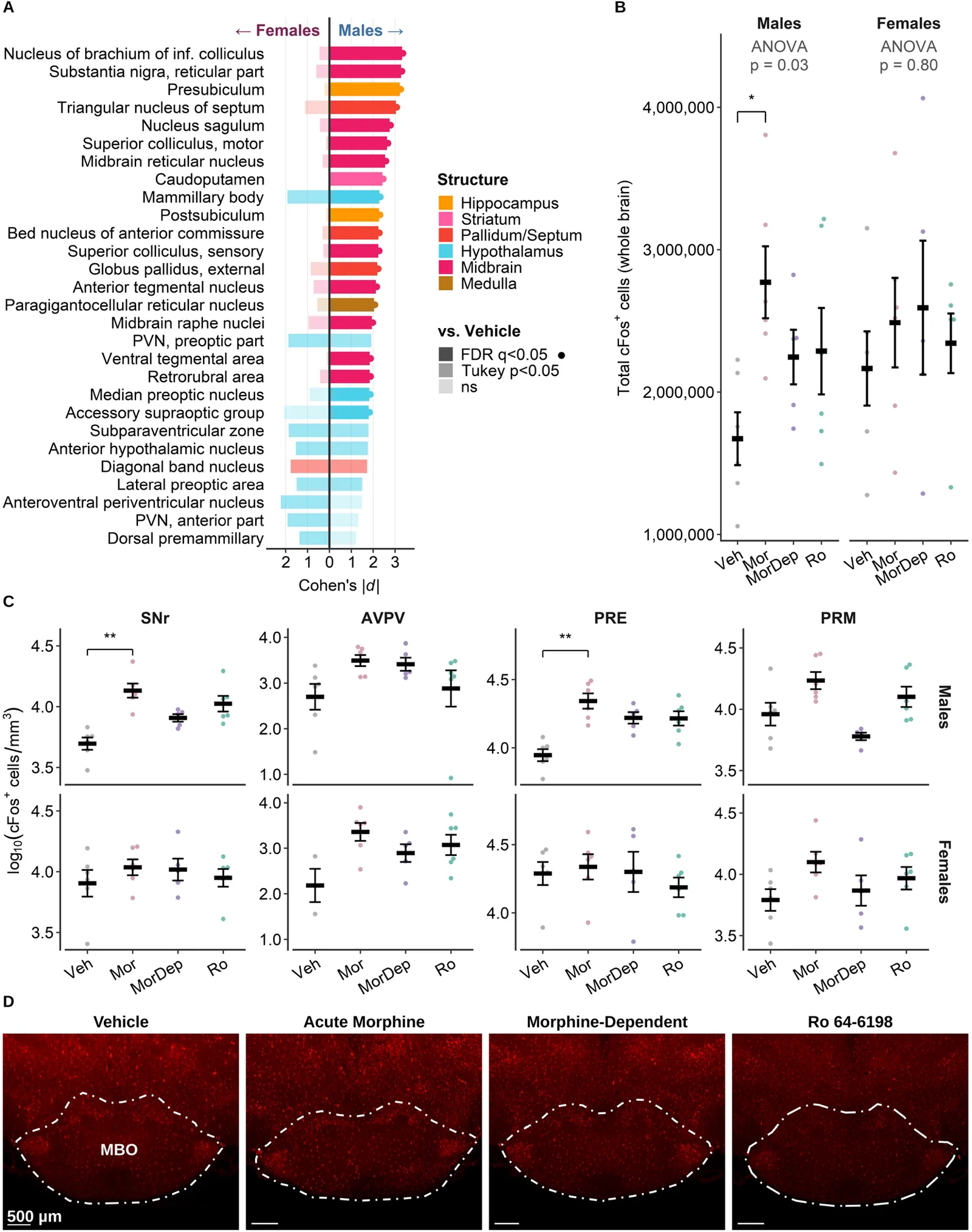
Regional activation differs by sex and is largely sustained under dependence. (A) Diverging bar plot of sex-stratified effect sizes (Cohen’s |d| vs. vehicle) for the 28 regions meeting either criterion (FDR q < 0.05 in either sex, or Tukey p < 0.05 in females), males (right) vs. females (left), colored by division; fill opacity indicates FDR q < 0.05, Tukey p < 0.05, or n.s. (B) Whole-brain total tdTomato^+^ (c-Fos-expressing) cell counts per condition, shown separately for males and females; each point is one animal, bars are mean ± SEM (one-way ANOVA: males p = 0.03, females p = 0.80). (C) Exemplar cFos density (log_10_ cells/mm^3^) for substantia nigra reticular part (SNr), anteroventral periventricular nucleus (AVPV), presubiculum (PRE), and paramedian lobule (PRM), split by sex across conditions; points are animals, mean ± SEM (*q < 0.05, **q < 0.01). (D) Representative light-sheet coronal images of tdTomato signal in the mammillary body (MBO) by condition; 500 μm scale bar. Per-sex n = 6/6/5/6.

**Figure 3.**
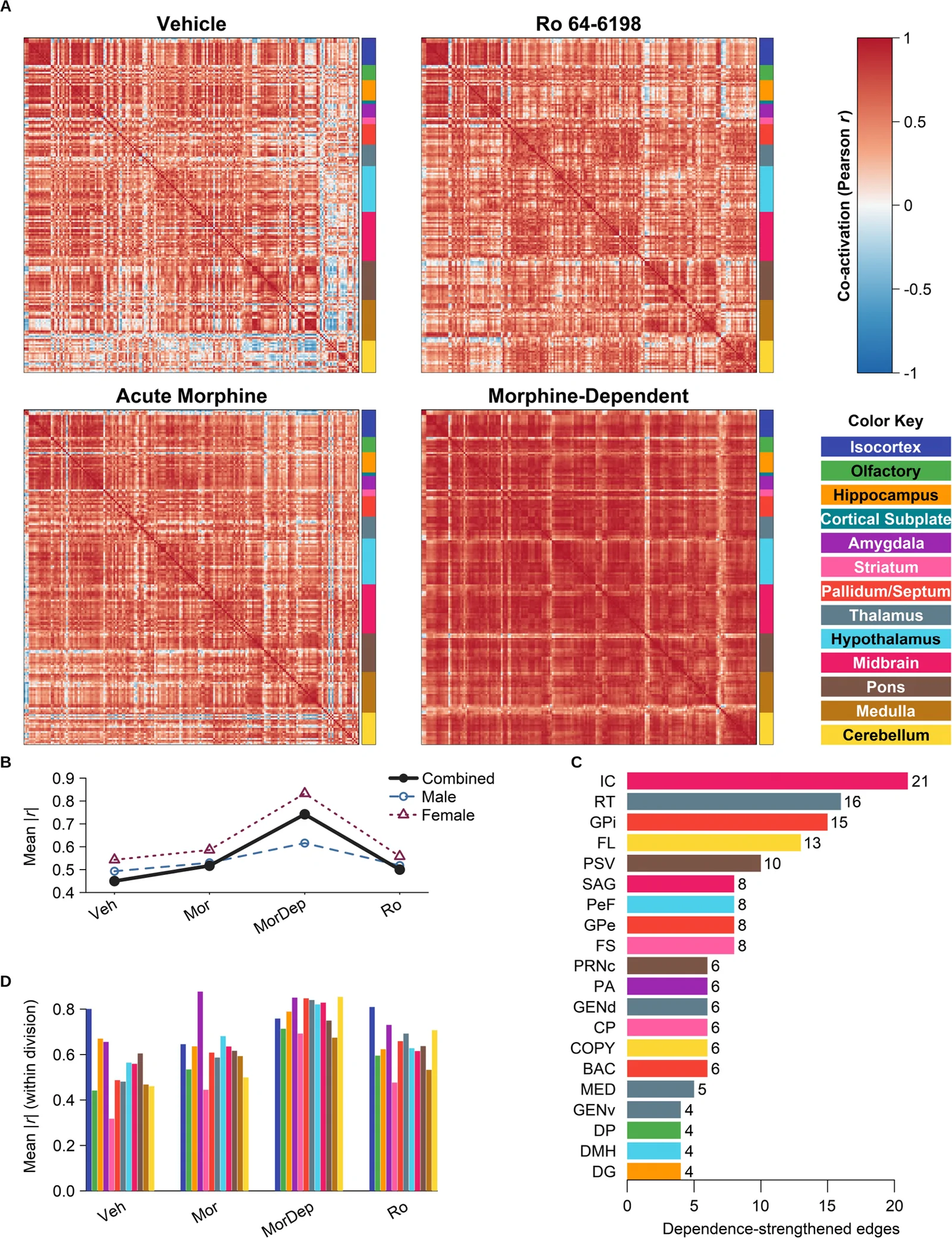
Elevated brain-wide co-activation under morphine dependence. (A) Region × region co-activation matrices (Pearson r across animals, 198 grey-matter regions) for each condition (Vehicle and Ro 64–6198, top; Acute Morphine and Morphine-Dependent, bottom); regions ordered by CCFv3 division (Color Key, right) with a shared correlation scale. (B) Mean absolute co-activation (mean |r|) per condition for the combined network and for each sex. (C) Region involvement among the 160 region pairs with significantly strengthened co-activation under dependence (Vehicle vs. Morphine-Dependent; Fisher r-to-z, BH-corrected), shown for the 20 most-involved regions and colored by division. Because each pair involves two regions, bars sum to more than the number of pairs. (D) Mean within-division co-activation (mean |r|) by division and condition. The Cortical Subplate is omitted from D (2 regions form a single within-division pair, insufficient for a stable mean), but still appears in the heatmap anatomy bars and Color Key. Combined-sex n = 12/12/10/12.

**Figure 4.**
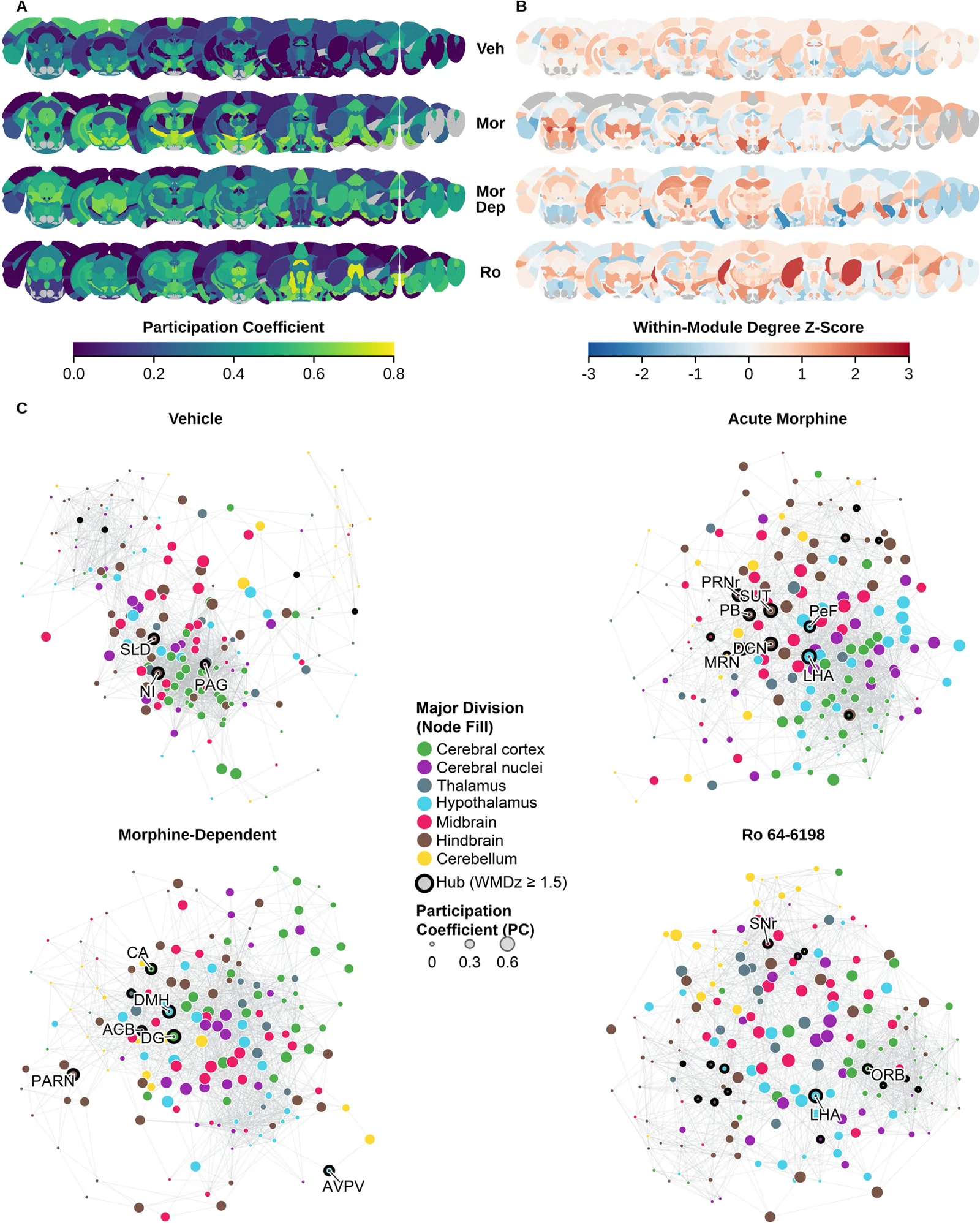
Network cartography and organization. (A) Participation coefficient (PC) mapped onto CCFv3 coronal sections for each condition (PC scale 0–0.8). (B) Within-module degree z-score (WMDz) on matched coronal sections, same conditions (scale −3 to 3). (C) Force-directed (Kamada–Kawai) representations of each co-activation network (density-matched at 10% edge density, 1,951 edges per network); node fill indicates major brain division, node size scales with PC, and black rings mark hubs (WMDz ≥ 1.5); labeled nodes are connector hubs. Layouts are rescaled per network, so inter-node distances are not quantitatively comparable across conditions. For node fill, the 13 CCFv3 divisions are collapsed to 7 major divisions (Isocortex, Olfactory, Hippocampus, and Cortical Subplate → Cerebral cortex; Amygdala, Striatum, and Pallidum/Septum → Cerebral nuclei; Pons and Medulla → Hindbrain; Thalamus, Hypothalamus, Midbrain, and Cerebellum retained). Combined-sex networks.

## Data Availability

All processed data needed to reproduce the analyses and figures, including the per-region cell-count and density matrices and the statistical and network result workbooks, are deposited in Zenodo (https://doi.org/10.5281/zenodo.22754844) [56]. All analysis code (R) is maintained in a public GitHub repository (https://github.com/toll-lab-code/cfos-opioid-brain-analyses) and archived in Zenodo (https://doi.org/10.5281/zenodo.22755396) [57]. The dataset and code are also cited in the reference list. Raw light-sheet imaging volumes are not deposited in a public repository owing to their size (on the order of terabytes per brain); this size exception is noted here, and the volumes are available from the corresponding author on reasonable request. The Allen Mouse Brain Common Coordinate Framework (CCFv3) is publicly available, and cell detection and atlas registration were performed in NeuroInfo (MBF Bioscience).
