# Supplementary material for: Whole Brain Cellular Activation After Mu and NOP Receptor Agonism Identifies Differential Regional and Network Consequences
Source: bioRxiv. 2026 Sep 28:2026.09.22.753526. Preprint. [Version 1] doi: 10.64898/2026.09.22.753526 (PMC13648335; doi:10.64898/2026.09.22.753526)
Supplement: Supplementary file 1 [file media-1.pdf]

Whole Brain Cellular Activation After Mu and NOP Receptor Agonism Identifies Differential Regional and Network Consequences

Supplementary Information

Supplementary Methods

Animals

TRAP2 (Fos2A-iCreER) and Ai9 (Rosa26-LSL-tdTomato) mice (The Jackson Laboratory, stocks #030323 and #007909) were crossed in-house to generate TRAP2/Ai9 reporter mice [1]. Male and female mice were used for all cohorts (n = 6 per sex per condition, except Morphine-Dependent, n = 5). Animals were 6–8 weeks of age at the start of the experiments, group-housed (3–5 per cage) under a 12 h light/dark cycle (lights on at 07:00) with food and water available ad libitum. All procedures were approved by the Florida Atlantic University IACUC and conducted in accordance with the NIH Guide for the Care and Use of Laboratory Animals.

Activity-dependent labeling and drug treatments

Neuronal activation was visualized genetically using TRAP2/Ai9 mice, in which tamoxifen inducible CreERT2 is expressed under the control of c-fos promoter. Administration of 4-hydroxytamoxifen (4-OHT) enables Cre-mediated recombination at the Ai9 locus [2], resulting in permanent tdTomato expression in neurons that were transcriptionally active within the labeling window [1, 3]. Mice were assigned to one of four conditions: Vehicle, Acute Morphine, Morphine-Dependent, or the nociceptin/orphanin FQ (NOP) receptor agonist Ro 64-6198. Morphine sulfate and Ro 64-6198 (both from the NIDA Drug Supply Program) were dissolved in vehicle (5% DMSO, 0.5% hydroxypropyl cellulose). Animals received a single drug administration for Acute Morphine (10 mg/kg, i.p.), Ro 64-6198 (0.6 mg/kg, i.p.) and vehicle 30

Commented [AO1]: this part needs to be described in Figure 1 or a supplemental figure

Commented [AO2]: paper for Ai9

Commented [AO3]: references: kasia's Pain paer and Greg corder one

min before 4-OHT injection. For Morphine-Dependent condition, animals received an escalating, twice-daily (a.m./p.m., approximately 8 h apart) morphine regimen over four days (Day 1, 10/15 mg/kg; Day 2, 20/30 mg/kg; Day 3, 50/60 mg/kg; Day 4, 70/75 mg/kg), followed on Day 5 by a single morning challenge dose (25 mg/kg, i.p.) 30 min before 4-OHT injection, to ensure animals were not in withdrawal. 4-OHT (50 mg/kg, i.p., in corn oil) [4] was administered 30 min after the drug/vehicle injection, at the period of peak drug effect, and mice were left undisturbed in the testing environment for 2 h. All animals were perfused 10 days after 4-OHT injection to allow robust tdTomato expression.

Commented [AO4]: approximate timing, like at least 6 hours between the injections etc.

The two morphine conditions differ in the dose administered during the labelling window as well as in treatment history. Acute-morphine animals were labelled following a single 10 mg/kg injection, whereas morphine-dependent animals were labelled following the 25 mg/kg Day 5 challenge, which was given to ensure that animals were not in withdrawal at the time of labelling. The design therefore does not separate treatment history from challenge dose, and no acute 25 mg/kg control group was included. Because dependent animals received the larger challenge dose yet showed lower activation than acutely treated animals across cerebellar, pontine and medullary structures, the reduction reported for those regions is a conservative estimate of the effect of chronic treatment; the same reasoning does not apply in the opposite direction, and regions showing comparable activation across the two conditions should not be read as evidence of preserved sensitivity.

Tissue clearing and light-sheet imaging

Commented [AO5]: Although you cite your previous paper here, please describe the complete methodological procedure for clearing, as a citation alone is not sufficient for readers to understand the approach.

Whole brains were cleared (not immunolabeled) using MDISCO, a fluorescence-preserving solvent-based clearing protocol [5], and imaged intact on a Miltenyi UltraMicroscope Blaze II light-sheet fluorescence microscope using uniform acquisition parameters across samples.

Mice were transcardially perfused with 20–30 mL of 1× PBS followed by 20–30 mL of freshly prepared, filtered 4% (w/v) paraformaldehyde (PFA) in PBS (pH 7.4); brains were dissected, post-fixed in 4% PFA overnight at 4 °C, and washed in 1× PBS overnight at 37 °C. Fixed brains were dehydrated through a graded methanol (MeOH) series in water (20%, 40%, 60%, and 80% v/v, 1 h each at room temperature [RT]) followed by two changes of 100% MeOH (1 h, then overnight at RT). Samples were delipidated and permeabilized in 66% dichloromethane (DCM)/33% MeOH (v/v) overnight at RT, returned to 100% MeOH (two 2-h washes at RT), and bleached in freshly prepared 6% hydrogen peroxide in MeOH (one part 30% H_2O_2 to four parts MeOH) overnight at 4 °C to reduce background. After post-bleaching MeOH washes (1 h, then overnight at RT), brains underwent a final delipidation in 66% DCM/33% MeOH (3 h at RT) and 100% DCM (1 h at RT). For refractive-index (RI) matching, samples were transferred to 100% dibenzyl ether (DBE) and rocked at RT until optically transparent, held in DBE for 4–5 days to allow internal bubbles to dissipate, and finally transferred to ethyl cinnamate (ECi) for final RI matching, imaging, and long-term storage. These MDISCO-specific modifications preserve endogenous tdTomato fluorescence and yield uniform optical transparency without antibody-based signal amplification. Tiled acquisitions were stitched into whole-brain volumes with MACS iQ View (Miltenyi Biotec). Stitched volumes were inspected in Imaris (Oxford Instruments) for reconstruction quality and signal uniformity and for preparation of representative images only (no quantitative measures were derived in Imaris).

Atlas registration, region cleaning, and quantification

Whole-brain volumes were processed in NeuroInfo [6] for automated cell detection, registration, and region-based quantification. Volumes were registered to the Allen Mouse Brain Common Coordinate Framework (CCFv3) by an intensity-based approach (coarse

manual alignment followed by nonlinear registration). tdTomato-positive somata were detected automatically using a size constraint of 10–35 μm diameter and assigned to CCFv3 structures; all registrations and detections were quality-reviewed before export.

NeuroInfo returns counts for all CCF structures, including white matter, fiber tracts, and ventricular spaces. The exported output was cleaned to the analyzed region set as follows: (i) only grey-matter regions were retained; (ii) left and right hemisphere counts were averaged to a single bilateral value per region; and (iii) the ventral-most regions, where the brain is adhered to the imaging stage and the mounting adhesive impedes light transmission and yields unreliable signal, were excluded. This yielded a final analyzed set of 198 grey-matter regions; the complete list of included and excluded regions is provided in Table S1. Region volumes used for density normalization were taken from the CCFv3 reference [7].

The a priori exclusions listed in Table S1 include several structures with established roles in opioid action. Among them are the nucleus raphe magnus, nucleus raphe pallidus and nucleus raphe obscurus, which together with adjacent reticular nuclei constitute the rostral ventromedial medulla; the arcuate, ventromedial and supraoptic hypothalamic nuclei; and the ventrolateral and ventromedial preoptic nuclei. These regions lie at or near the ventral surface, where the mounting adhesive impedes light transmission, and were excluded on that technical basis rather than on any anatomical or functional criterion. Regional and network results should accordingly be read as describing the 198 analysed regions rather than the whole grey-matter brain. The descending pain-modulatory pathway in particular is represented in the analysed set by the periaqueductal grey, pedunculopontine nucleus and pontine reticular nuclei, but not by the rostral ventromedial medulla itself, and the hypothalamic findings

reported here are drawn from the 27 hypothalamic regions that survived exclusion rather than from the full hypothalamus.

Regions were assigned to anatomical divisions following the Allen Mouse Brain Common Coordinate Framework (CCFv3) ontology, with two functional groupings: amygdalar nuclei were treated as a single Amygdala division rather than divided between the cortical subplate and striatum as in the strict CCFv3 hierarchy, and the lateral septal complex was grouped with the pallidum (Pallidum/Septum). The amygdalar grouping reflects the functional coherence of the amygdalar complex, which is recruited as a unit in drug-related brain-wide co-activation [8]. It affects only the anatomical color-coding and the descriptive within-division co-activation summary (Figure 3D), not the regional statistical models or the network metrics, which operate at the region and module level. The brainstem was resolved into separate Pons, Medulla, and Cerebellum divisions, yielding 13 anatomical divisions across the 198 analyzed regions. For the force-directed (Kamada-Kawai) [9] network graphs (Figure 4), the 13 anatomical divisions were collapsed to seven major Allen divisions (Cerebral cortex: isocortex, olfactory, hippocampus, and cortical subplate; Cerebral nuclei: amygdala, striatum, and pallidum/septum; Thalamus; Hypothalamus; Midbrain; Hindbrain: pons and medulla; and Cerebellum) for legibility, since 13 node-fill colors are not reliably distinguishable in the dense graph layouts. All other panels, including the correlation heatmaps and the cartographic role plots, retain the full 13-division scheme.

Normalization and regional statistical models

Total brain-wide tdTomato+ cell counts were compared across the four conditions by one-way ANOVA. Per-region tdTomato+ neuron counts were exported separately for males and females. Counts were converted to density (cells/mm³) using CCFv3 region volumes [7], with

zero values set to missing prior to $\log_{10}$ transformation. Because $\log_{10}(0)$ is undefined, animals with a zero count in a region were excluded from that region's ANOVA (1 value in males and 17 in females, across 11 regions), with the number of animals entering and excluded per region reported in the deposited workbooks. The primary regional test was a one-way ANOVA on $\log_{10}$ density with condition as the sole factor and males and females pooled, followed by Tukey-adjusted pairwise contrasts for the five a priori comparisons; p-values were corrected across the 198 regions by the Benjamini-Hochberg procedure [10]. The Morphine-Dependent versus Ro 64-6198 contrast, the sixth possible pairwise comparison, was excluded a priori because these conditions differ simultaneously in receptor target (mu versus NOP) and in treatment history (chronic versus acute), so it confounds the two factors and does not test an interpretable hypothesis. As secondary analyses, the one-way ANOVA was repeated within each sex (males and females analyzed separately). A condition × sex two-way ANOVA on $\log_{10}$ density was run as a supplemental analysis, testing the sex main effect and the condition × sex interaction per region (each Benjamini-Hochberg corrected across regions) alongside the condition contrasts. For each comparison we report Cohen's d (computed on the $\log_{10}$-density scale) and the Benjamini-Hochberg FDR q-value from the pooled, male-only, and female-only tests and from the negative-binomial GLM robustness model, together with the omnibus ANOVA F and its FDR q and the condition × sex (sex main-effect and interaction) q-values (Table S2). Benjamini-Hochberg FDR ($q < 0.05$) is the inferential criterion throughout; the sex-stratified ANOVAs are secondary/exploratory, and where FDR returned no significant regions within a sex-stratified panel, Tukey-adjusted p-values (not corrected across regions) are shown descriptively only — explicitly flagged as not surviving FDR correction — rather than as inferential claims.

Model assumptions were assessed per region: residuals departed from normality (Shapiro-Wilk $p < 0.05$) in 63 of 198 regions, whereas variances were homogeneous in all but 8 (Brown-Forsythe $p < 0.05$). As a quality-control check on the primary log-density ANOVA, a negative binomial GLM was fit per region on bilateral summed counts (hemisphere averages × 2; MASS::glm.nb) with a condition × sex design and log(region volume) as an offset, using a likelihood-ratio omnibus test and Tukey-adjusted emmeans contrasts on the condition factor. This count-based model does not assume log-normally distributed densities and accommodates overdispersion; it is reported as a robustness check rather than the primary analysis and was used to confirm that the direction and significance of the primary findings are not driven by the distributional assumptions of the ANOVA.

Network construction

For each condition × sex, region × animal matrices were cleaned by setting negative values to missing and imputing zeros with half the minimum nonzero value for that animal (a commonly used substitution for log transformation of count/omics data) [11], followed by $\log_{10}$ transformation. This differs from the regional analysis, where zeros are excluded rather than imputed, because network construction requires a complete value for every region in every animal to compute pairwise correlations, whereas a per-region ANOVA can simply omit the missing observation. Regions with no variance or no valid data were dropped. Interregional co-activation was quantified as the Pearson correlation between regions across animals.

The primary analysis used a pooled Combined network. Because the per-sex sample size ($n = 5–6$) is too small to estimate stable interregional correlations, males and females were pooled after mean-centering each region within sex [12], which removes the between-sex mean shift that would otherwise induce spurious cross-region correlations while preserving the

within-sex covariance the network relies on, and provides the sample size (n = 10–12) needed for stable network estimation and for bootstrap and permutation inference. Sex-stratified (Males, Females) networks were computed as a supplemental sensitivity analysis (Tables S3–S5); consistent with their limited power at this sample size they yielded few differentially co-activated pairs, and sex differences are examined at the regional level (Figure 2) rather than the network level.

Thresholding

Networks were proportionally (density-matched) thresholded by retaining the strongest positive correlations until a fixed proportion of possible edges survived, set to a common density of 10% across all conditions so that degree-, modularity-, and role-based metrics are compared at matched density rather than confounded by condition differences in overall correlation strength [13, 14]. Thresholding fixed the edge count identically across networks (1,951 edges); regions left with no suprathreshold edge were then excluded, so density among the retained regions ranged from 0.101 to 0.121 rather than a uniform 0.10. Only positive edges were retained [15, 16]. The heatmaps in Figure 3A display the full correlation matrix, including negative values, whereas all graph metrics are computed on the positive-edge thresholded network. This proportional scheme is a departure from the fixed absolute-r threshold common in the c-Fos co-activation literature and is adopted to ensure cross-condition comparability of the graph metrics; mean absolute correlation (|r|) per condition is reported separately so that the proportional-thresholding caveat can be assessed.

Module detection

Communities were detected directly on the thresholded weighted graph by consensus Louvain (1,000 weighted-Louvain runs; co-classification matrix thresholded at 0.5 and re-

clustered to convergence) [17], which addresses Louvain stochasticity and the modularity resolution limit and provides a stable, graph-native partition for the cartographic metrics. To document the basis for this choice, we also computed module counts using the standard c-Fos approach (Euclidean distance between regions' interregional-correlation profiles, complete-linkage hierarchical clustering, dendrogram cut at 0.3–0.7 of maximum tree height) [8, 18] on the full correlation matrix; because a single tree cut depends on the chosen cut height and is subject to the modularity resolution limit, these counts were computed only as a comparison with the standard approach rather than as the basis for the cartographic analysis, for which the consensus-Louvain partition was used.

Node roles and hubs

Cartographic node roles were assigned from the within-module degree z-score (WMDz) and participation coefficient [19], computed on the (thresholded graph, consensus-module) pair. Roles were driven by the weighted WMDz (a unit-SD within-module z-score on this data), with the hub/non-hub boundary set at WMDz = 1.5, the lower bound of the canonical high-WMDz range [20]. We note that the value of 0.8 reported in the c-Fos lineage was derived from a compressed WMDz distribution and is not comparable to a unit-SD z-score; on these data 0.8 over-calls hubs (~25% of nodes), whereas 1.5 flags roughly 4% of nodes (~8 hubs per network). Both binary and weighted WMDz/participation variants are reported; weighted variants drive the role assignment. In this scheme, hubs are further distinguished by participation coefficient into provincial hubs (edges concentrated within their home module) and connector hubs (edges distributed across modules); the between-module connector hubs are the regions labeled in each network in Figure 4C, with the multi-centrality convergence below reported as independent supporting evidence. Roles for regions in modules smaller than

five nodes are flagged as lower-reliability (small-module instability and participation-coefficient size bias) [21].

As convergent evidence independent of the modular partition, hubs were also identified by agreement across four centrality measures (strength, weighted betweenness, weighted closeness, and eigenvector centrality) [22, 23]. A region was scored "high" on a measure if it exceeded the within-network mean by more than one standard deviation and was classified a hub if high on at least two of the four measures. Eigenvector centrality was computed on the giant connected component, with off-component nodes set to missing; because the sex-stratified networks fragment into multiple components at matched density, this guard was necessary. A continuous hub-ness score (mean within-network z across the four centralities) is also reported for ranking.

Commented [DZ6]: This is a very nice piece of work!

Global metrics and modularity significance

Global metrics (mean strength, weighted transitivity, global efficiency, and Louvain modularity Q) were computed on the density-matched graph after isolate removal, with the number of components and giant-component fraction reported. Modularity significance was tested against a degree-preserving (configuration-model) null in which the observed graph was rewired preserving each node's degree (Maslov-Sneppen edge swaps) [13]; we report observed Q, the null mean and SD, a modularity z-score, and a permutation p-value (5,000 permutations). A small-world index [24] was computed against 100 randomly rewired nulls (each-edge rewiring, which does not preserve the degree sequence).

Commented [DZ7]: Also a very good way to do this. It is very clear you've done your homework (hopefully it will be to reviewers as well)

Co-activation differences

Edge-wise condition differences in interregional co-activation were tested by Fisher r-to-z transformation (two-sided), with Benjamini-Hochberg correction across all region pairs, for the

same five a priori condition contrasts; the per-pair correlation difference (Δr) is reported alongside. Co-activation structure is displayed per condition as full region × region correlation heatmaps, with regions ordered by anatomical division and decoded by a division color key (Figure 3A). Overall co-activation strength is summarized as the mean absolute correlation (|r|) per condition (Figure 3B), and within-division co-activation as the mean |r| among within-division region pairs for each division across the four conditions (Figure 3D); anatomical divisions with fewer than three regions (the cortical subplate) are omitted from the within-division summary. The number of region pairs strengthened under dependence is ranked by region (Figure 3C). These heatmaps and division summaries are descriptive; inferential testing of condition differences in co-activation is provided by the edge-wise Fisher r-to-z analysis above.

Sensitivity of co-activation strength to animal-level variance

Because interregional co-activation is computed across animals, any component of variance shared by all regions within an animal — for example, animal-to-animal differences in overall labelling efficiency — contributes to every region pair and therefore raises the mean absolute correlation independently of regional coordination. We quantified this by decomposing each condition's region × animal matrix into a per-animal global offset (the mean $\log_{10}$ density across regions) and the region-specific residual. The proportion of variance attributable to the global offset was 27.2% under vehicle, 41.7% under acute morphine, 62.6% under morphine dependence and 33.9% under Ro 64-6198. The elevated value in dependent animals reflects reduced region-specific variance (SD 0.123, versus 0.294 under vehicle) rather than increased global variance, which was comparable across conditions (SD 0.167 versus 0.188). Removing the per-animal offset before computing correlations reduced mean |r| to 0.397 (vehicle), 0.320

(acute morphine), 0.369 (morphine-dependent) and 0.365 (Ro 64-6198); median-centring and global-signal regression of the same matrices gave equivalent results. The increase in co-activation under dependence therefore reflects a shift in the ratio of animal-level to region-specific variance — that is, activation profiles that are more stereotyped across dependent animals — as much as an increase in coupling between specific region pairs, and the two cannot be separated at this sample size. Uncorrected values are reported throughout for comparability with the c-Fos co-activation literature, in which per-animal normalisation is not applied [8, 18], and the dependence effect is interpreted accordingly.

Inference on network metrics

Because each condition yields a single correlation network, global metrics are point estimates without intrinsic error bars. To support inferential claims, we resampled over animals (the unit of replication) on the Combined networks: (i) bootstrap confidence intervals were obtained by resampling animals with replacement, rebuilding the network at matched density, and recomputing each metric (1,000 iterations), yielding a 95% CI per condition; and (ii) between-condition differences were tested by label-permutation, pooling animals, shuffling condition labels at the observed group sizes, and recomputing the metric difference under the null (two-sided p; 1,000 permutations) [8, 25]. With small samples, bootstrap resampling can duplicate animals and produce wide intervals; this width reflects the sample size honestly. Benjamini-Hochberg correction was applied within two metric families analyzed separately: a Strength family (mean strength, which re-expresses co-activation strength at matched density) and a Topology family (weighted transitivity, global efficiency, modularity Q), mirroring the lineage's separation of correlation strength from graph topology [8, 26].

Software and packages

Analyses were performed in R 4.4.3. Regional models used readxl, openxlsx, dplyr, MASS, and emmeans; network analyses used tidyverse and igraph. Figures used ggplot2 with showtext, ggbeeswarm, and scales (regional figures); base R graphics (network co-activation heatmaps, division summaries, and force-directed network graphs); and jsonlite, xml2, rsvg, magick, and viridisLite (atlas projections). Coronal section geometry for the atlas projections (Figure 4 A, B) is from the Scalable Brain Atlas template for the Allen Mouse Brain Common Coordinate Framework v3 (ABA_v3) [27, 28]; participation coefficient and WMDz were matched to atlas regions by acronym, and regions without a value in a given condition are shown in grey. All stochastic procedures (consensus Louvain, modularity permutation, bootstrap and label-permutation resampling, and the force-directed graph layout) were run under fixed random seeds, so the deposited code reproduces the reported values exactly. Results were exported as topic-grouped Excel workbooks (00_Processed_Data, 01_Connectivity, 02_Network_Metrics, 03_Modules_and_Roles) and the regional statistical workbooks (Primary, BySex, TwoWay, NegBin).

AI use statement

Generative AI (Claude, Anthropic; model Opus 4.8) was used during manuscript preparation and in preparing the analysis code for public deposit. Specifically, it was used to: (i) correct, update, and debug analysis code written by the authors, and to write utility scripts used to assemble the supplemental tables; (ii) consistency-check manuscript and supplemental text, including terminology standardization and cross-checking reported numerical values against the source data workbooks; and (iii) prepare the deposited R code for public release, including removing machine-specific file paths, generalizing input handling, standardizing comments and headers, and drafting repository documentation. The study design, the analytical approach, the

execution of all statistical and network analyses, and the interpretation of results were determined and carried out by the authors. No generative AI image tools were used to create figure content; all figure content was rendered directly from the study data by R and Python scripts. All AI-assisted outputs were reviewed, verified, and where necessary corrected by the authors, who take full responsibility for the accuracy and integrity of the work.

Supplementary Results

Pooled Regional Analysis

Results presented here are regional data pooled from male and female mice. Individual sex data is presented in the main body of the manuscript. Across the four conditions (vehicle, acute morphine, morphine-dependent, and Ro 64-6198), 84 regions showed elevated c-Fos+ density under acute morphine relative to vehicle at Tukey $p < 0.05$ (all Vehicle < Morphine), of which 44 survived Benjamini–Hochberg correction ($q < 0.05$; Table S2). The strongest effects were concentrated in the periventricular hypothalamus, preoptic area, and adjacent basal forebrain (all $|d| > 1.5$, Figure S1A), led by the mammillary body (MBO; $F(3,42) = 13.11$, $p = 3.5 \times 10^{-6}$; Tukey Vehicle vs. Morphine $q = 0.002$, $|d| = 2.14$, exemplified in Figure S1D) and including the accessory supraoptic group, periventricular and subparaventricular nuclei, diagonal band nucleus, and medial and lateral preoptic areas (Figure S1A). Representative light-sheet images show the elevated tdTomato+ signal in the mammillary body (MBO) under acute morphine relative to vehicle (Figure 2D). A secondary cluster spanned midbrain raphe, lateral lemniscus, and substantia nigra reticulata, with limbic extension through medial and basomedial amygdala and piriform cortex. The full ranked set, with FDR q-values, is reported in Table S2. Refitting the combined-sex comparisons with a negative binomial GLM on bilateral summed counts replicated 43 of the 44 FDR-significant regions under acute morphine, with 99.5% sign agreement across all 198 regions (e.g., MBO $q = 2.2 \times 10^{-6}$), confirming the effects were not an artifact of the $\log_{10}$ transformation. Despite morphine's Gi/o coupling, no region showed a significant decrease in tdTomato expression. A breakdown of the number of significant and FDR-significant differences from vehicle for each treatment group is shown in Figure S1B.

Following four days of escalating morphine (Figure 1B), the combined-sex analysis identified 49 regions at Tukey $p < 0.05$ versus vehicle, of which 15 survived FDR correction (Table S2). The periventricular-preoptic core identified under acute morphine persisted under dependence — MBO

(Tukey Vehicle vs. Morphine-Dependent $q = 0.008$, $|d| = 2.06$), accessory supraoptic group, medial preoptic and anterior hypothalamic nuclei (all FDR-significant). Every region significant under dependence was also significant under acute morphine: the 15 FDR-significant regions formed a strict subset of the 44 engaged acutely, and no region crossed the FDR threshold uniquely under dependence. Consistent with the two morphine conditions not differing after correction on direct comparison (below), dependence sustained a subset of the acute-morphine activation rather than recruiting a distinct regional profile. Additional forebrain and striato-limbic regions (lateral septum, accumbens, basolateral amygdala, anterior cingulate) reached significance (Tukey $p < 0.05$) only, accompanying the persistent FDR-significant core (Table S2).

Robustness of the regional analysis

Two sensitivity models were fitted alongside the primary $\log_{10}$-density ANOVA. The first, a negative binomial GLM on bilateral summed counts with log(region volume) as an offset, relaxes the log-normality assumption of the primary model. The second re-ran the one-way ANOVA on globally normalised densities, in which each region is expressed relative to that animal's brain-wide activation, and therefore asks whether a region is engaged disproportionately rather than in absolute terms. Each is more permissive than the primary analysis for some contrasts and more conservative for others. Both are reported here as sensitivity analyses; Benjamini–Hochberg-corrected inference throughout the manuscript is based on the primary ANOVA, which is the more conservative model.

Both models reproduced the principal regional findings. The negative binomial GLM returned more FDR-significant regions than the primary ANOVA for the vehicle contrasts (93 versus 44 under acute morphine; 35 versus 15 under dependence), replicating 43 of the 44 acute-morphine regions with 99.5% sign agreement across all 198 regions. Global normalisation, which by construction removes any brain-wide change in activation, left only the accessory supraoptic group and the mammillary body FDR-

significant under acute morphine, both of them among the 44, consistent with the acute-morphine increase being predominantly global rather than regionally selective. Under dependence, by contrast, 12 regions remained FDR-significant after global normalisation, 11 of them among the 15 identified by the primary analysis, indicating that the periventricular–preoptic core is engaged disproportionately and not merely as part of a brain-wide rise.

The two sensitivity models converge on a single point of difference from the primary analysis. For the acute morphine versus morphine-dependent contrast, where the primary ANOVA identified no region surviving FDR correction, the negative binomial GLM identified seven regions (COPY, PRM, PYR, UVU, AT, NLL and ECU) and global normalisation identified four (COPY, PRM, UVU and AT, a subset of the seven), in every case with lower activation in dependent animals and in every case a cerebellar, pontine or tegmental structure. These are the same regions described above as showing lower c-Fos+ density in dependent animals at Tukey $p < 0.05$. Because the primary $\log_{10}$-density ANOVA is the more conservative model, this reduction is reported throughout as a non-significant trend; that two independently specified sensitivity models identify it nonetheless suggests it is unlikely to be noise, and it is flagged here for that reason.

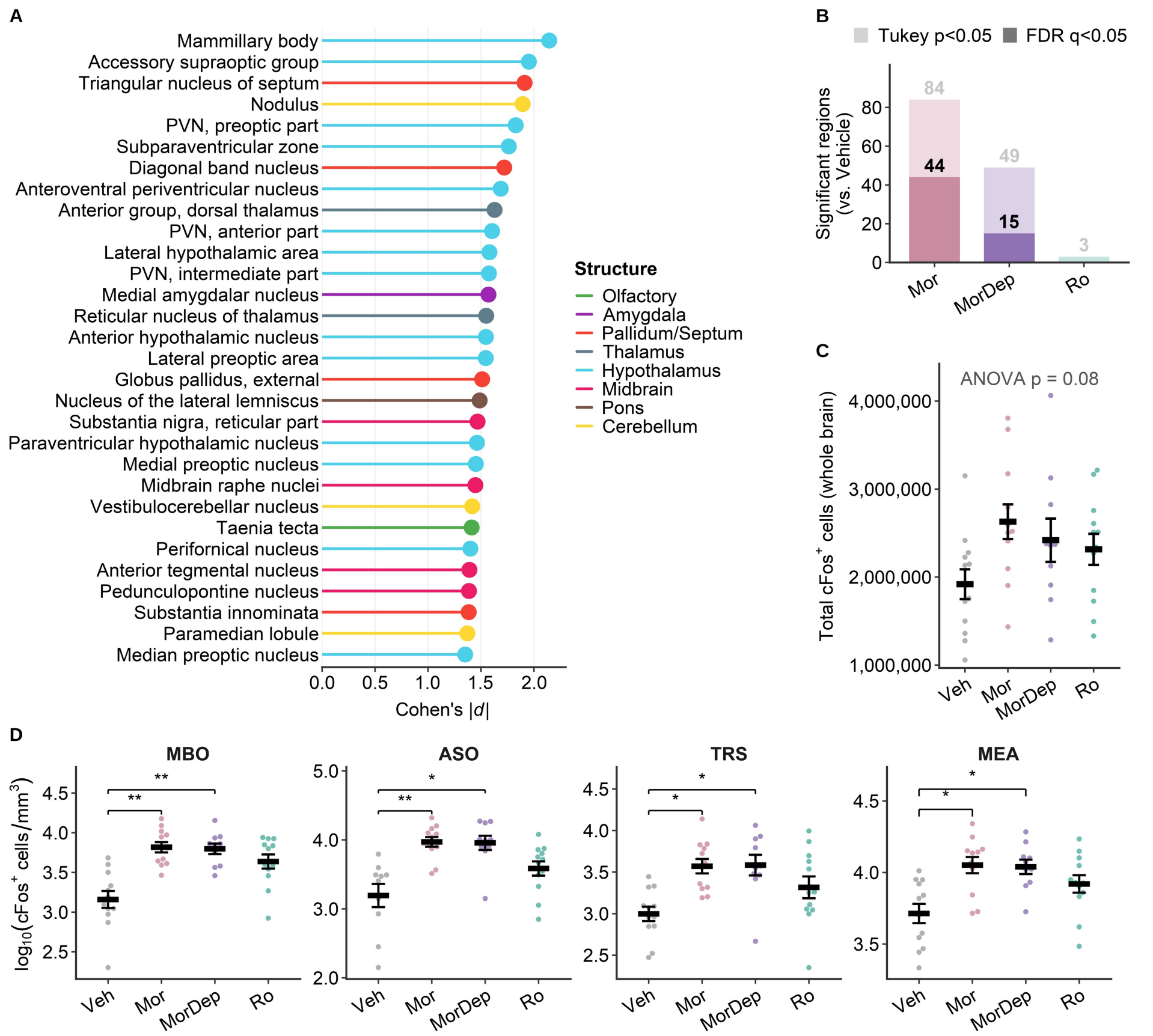

Figure S1. Whole-brain regional activation after opioid and NOP receptor agonists (combined sexes). (A) Lollipop plot of the 30 top-ranked regions by acute-morphine effect size (Cohen's |d| vs. vehicle), ordered and colored by CCFv3 division. (B) Number of regions differing from vehicle for each treatment at Tukey $p < 0.05$ (light) and after Benjamini–Hochberg FDR correction, $q < 0.05$ (dark). (C) Whole-brain total tdTomato^{+} (c-Fos-expressing) cell counts per condition; each point is one animal, bars are mean ± SEM (one-way ANOVA, $p = 0.081$). (D) Exemplar c-Fos density ($\log_{10}$ cells/mm^3) for the mammillary body (MBO), accessory supraoptic group (ASO), triangular nucleus of septum (TRS), and medial amygdala (MEA); points are individual animals, mean ± SEM; brackets show Tukey-adjusted comparisons vs. vehicle (*$q < 0.05$, **$q < 0.01$). Combined-sex n = 12/12/10/12 (Vehicle/Acute Morphine/Morphine-Dependent/Ro 64-6198).

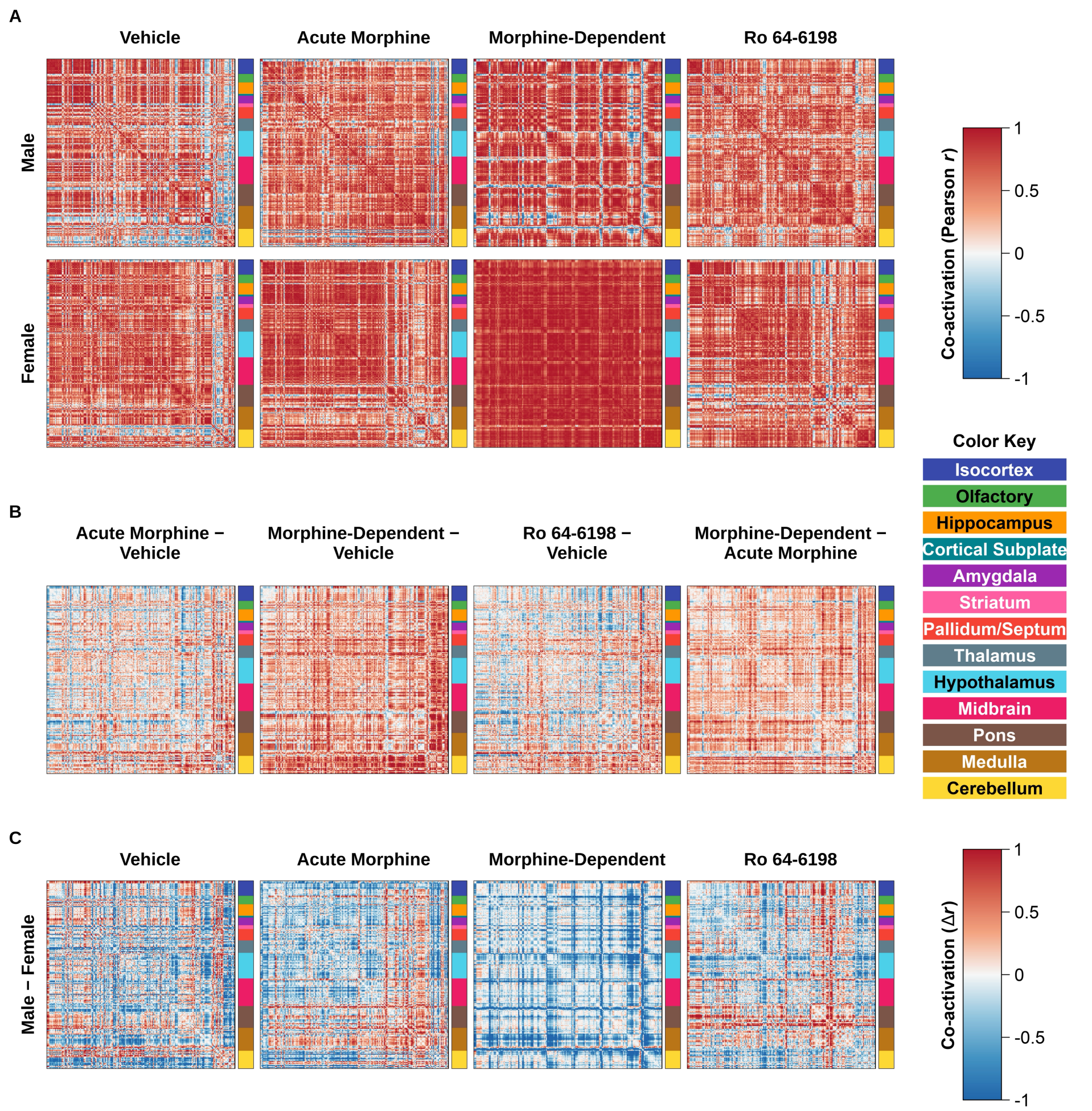

Figure S2. Sex-stratified whole-brain co-activation and its modulation across opioid and NOP-receptor conditions. Co-activation was quantified as the pairwise Pearson correlation of log_{10}-transformed regional c-Fos+ cell density across animals, over the 198 grey-matter regions. In every matrix, regions are ordered by anatomical division; the 13 major CCFv3 divisions are marked by the color bands flanking each matrix and defined in the Color Key. Conditions are Vehicle, Acute Morphine (10 mg/kg), Morphine-Dependent (escalating twice-daily dosing with a day-5 challenge), and Ro 64-6198 (0.6 mg/kg, NOP agonist); n = 6 per sex per condition except Morphine-Dependent (n = 5 per sex).

(A) Co-activation matrices for each condition (columns) in males (top row) and females (bottom row). Color encodes Pearson r from −1 to 1 (scale, top right).

(B) Between-condition differences in co-activation, computed as the element-wise difference (Δr) of the combined matrices (sexes pooled after within-sex mean-centering), in the treatment-minus-comparator direction: Acute Morphine − Vehicle, Morphine-Dependent − Vehicle, Ro 64-6198 − Vehicle, and Morphine-Dependent − Acute Morphine. Red indicates stronger co-activation in the first-named condition, blue in the comparator (Δr scale, bottom right). Sign convention matches Figure 3 (treatment − Vehicle, increase-positive).

(C) Sex differences in co-activation for each condition, computed as males − females. Red indicates stronger co-activation in males, blue in females (same Δr scale as B).

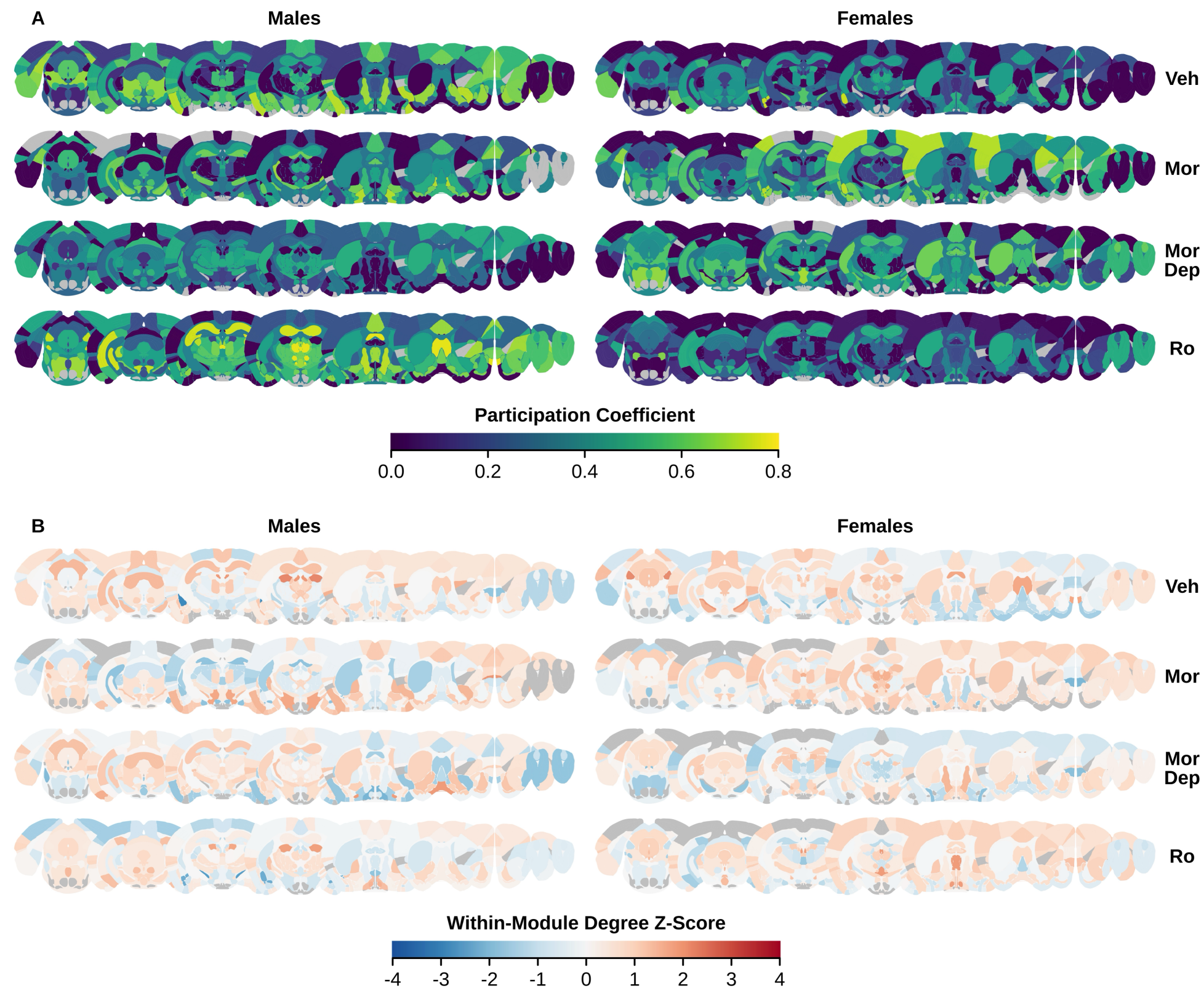

Figure S3. Sex-stratified cartographic node metrics across the whole brain. Participation coefficient (A) and within-module degree z-score (WMDz) (B) for each condition (rows: Vehicle, Acute Morphine, Morphine-Dependent, Ro 64-6198), shown separately for males (left) and females (right). Metrics were computed on the sex-stratified co-activation networks (n = 6 per sex per condition except Morphine-Dependent, n = 5 per sex; 10% edge-density threshold; consensus-Louvain modules) and projected onto nine coronal sections spanning anterior to posterior, using Scalable Brain Atlas geometry for the Allen Mouse Brain Common Coordinate Framework v3 (ABA_v3). Each atlas polygon is filled by the value of the region it represents, resolved by exact acronym match, then by averaging descendant regions present in the data, then by the nearest ancestor present in the data. Participation coefficient is shown on a viridis scale from 0 to 0.8 (A) and WMDz on a diverging blue–white–red scale from −4 to 4 (B). Polygons are left grey where no value could be resolved. This occurs in three cases: the region lies outside the 198 analyzed grey-matter regions (for example fiber tracts and ventricles); the region was excluded before network construction because its activation density had zero variance or no valid data in that condition and sex; or the region became isolated at the 10% density threshold and was removed from the graph. Grey coverage therefore differs between conditions and between sexes.

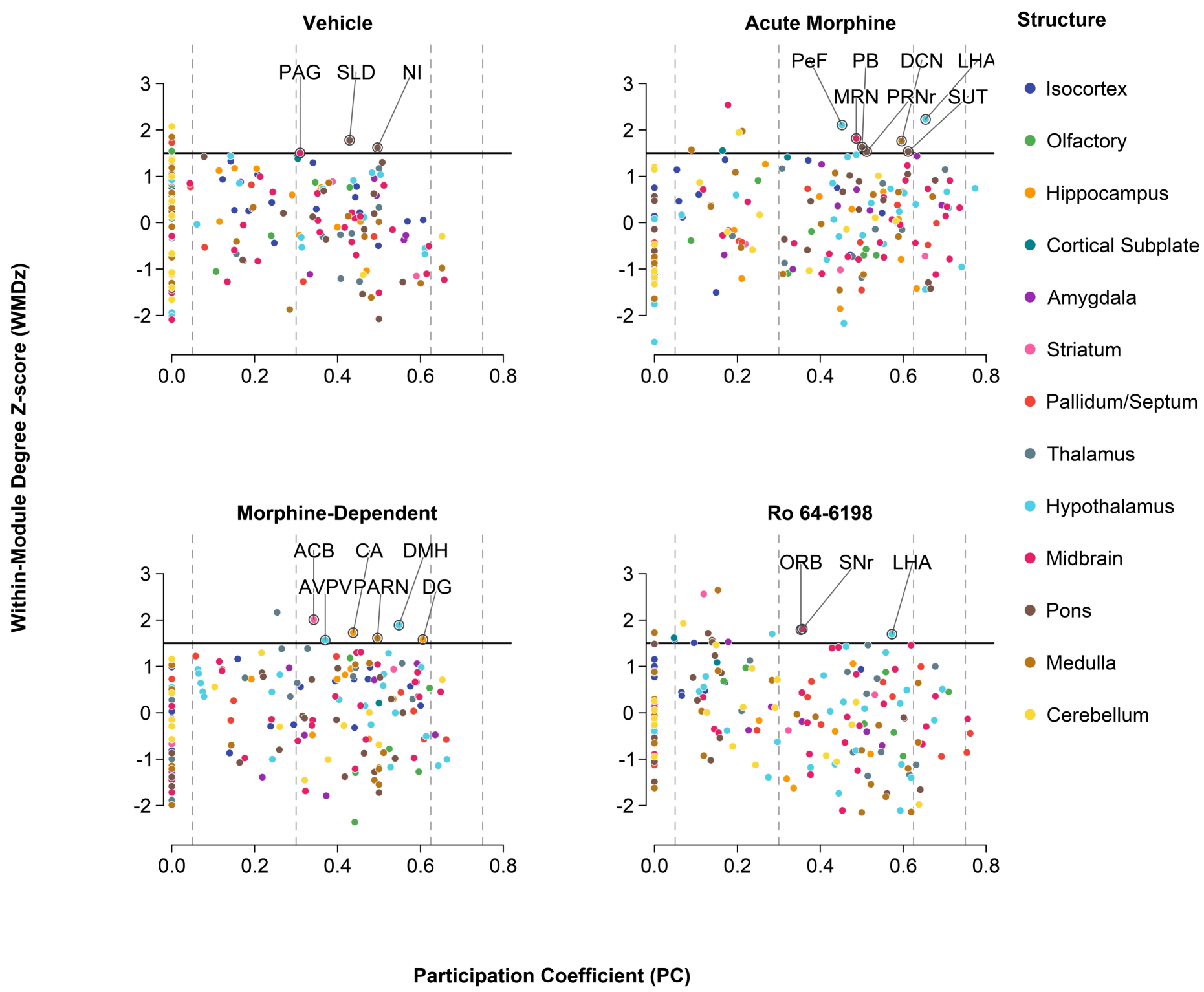

Figure S4. Cartographic node roles in the combined whole-brain co-activation networks. Guimerà–Amaral cartography for each condition (Vehicle, Acute Morphine, Morphine-Dependent, Ro 64-6198), derived from the combined, sex-pooled co-activation networks (within-sex mean-centered regional activation densities pooled across sexes; n = 12 per condition, n = 10 for Morphine-Dependent; 10% edge-density threshold; consensus-Louvain modules). Each point is one grey-matter region, positioned by its participation coefficient (PC, x-axis) and within-module degree z-score (WMDz, y-axis) and colored by major brain division (Structure key). The within-module degree z-score expresses how strongly each region is connected to other regions within its own module, in standard deviations relative to the other members of that module; the solid horizontal line marks WMDz = 1.5, the hub threshold (the lower bound of the canonical high-WMDz range; see Supplementary Methods). Regions above this line are hubs within their module. Dashed vertical lines mark the participation-coefficient boundaries of the Guimerà–Amaral role scheme, which subdivides hubs by how widely their connections are distributed across modules. As in Figure 4, only connector hubs (R6; WMDz ≥ 1.5 with intermediate participation, 0.30 < PC ≤ 0.75) are labeled by region abbreviation, whereas provincial hubs (WMDz ≥ 1.5, PC ≤ 0.30) and non-hub nodes are shown but unlabeled. Region abbreviations follow Allen CCFv3 nomenclature.

SI References

1. Targowska-Duda KM, Peters D, Marcus JL, Zribi G, Toll L, Ozawa A. Functional and anatomical analyses of active spinal circuits in a mouse model of chronic pain. Pain. 2024;165:685–697.

2. Madisen L, Zwingman TA, Sunkin SM, Oh SW, Zariwala HA, Gu H, et al. A robust and high-throughput Cre reporting and characterization system for the whole mouse brain. Nat Neurosci. 2010;13:133–140.

3. Corder G, Ahanonu B, Grewe BF, Wang D, Schnitzer MJ, Scherrer G. An amygdalar neural ensemble that encodes the unpleasantness of pain. Science. 2019;363:276–281.

4. Allen WE, DeNardo LA, Chen MZ, Liu CD, Loh KM, Fenno LE, et al. Thirst-associated preoptic neurons encode an aversive motivational drive. Science. 2017;357:1149–1155.

5. Martinez M, Thornberry J, Ozawa A, Toll L. MDISCO: a high-throughput tissue-clearing protocol for preservation of endogenous fluorescence in whole mouse brains. Bio Protoc. 2026;16:e5649.

6. Tappan SJ, Eastwood BS, O'Connor N, Wang Q, Ng L, Feng D, et al. Automatic navigation system for the mouse brain. J Comp Neurol. 2019;527:2200–2211.

7. Wang Q, Ding SL, Li Y, Royall J, Feng D, Lesnar P, et al. The Allen Mouse Brain Common Coordinate Framework: a 3D reference atlas. Cell. 2020;181:936–953.e20.

8. Kimbrough A, Lurie DJ, Collazo A, Kreifeldt M, Sidhu H, Macedo GC, et al. Brain-wide functional architecture remodeling by alcohol dependence and abstinence. Proc Natl Acad Sci U S A. 2020;117:2149–2159.

9. Kamada T, Kawai S. An algorithm for drawing general undirected graphs. Inf Process Lett. 1989;31:7–15.

10. Benjamini Y, Hochberg Y. Controlling the false discovery rate: a practical and powerful approach to multiple testing. J R Stat Soc B. 1995;57:289–300.

11. Wei R, Wang J, Su M, Jia E, Chen S, Chen T, et al. Missing value imputation approach for mass spectrometry-based metabolomics data. Sci Rep. 2018;8:663.

12. Alexander-Bloch A, Giedd JN, Bullmore E. Imaging structural co-variance between human brain regions. Nat Rev Neurosci. 2013;14:322–336.

13. Rubinov M, Sporns O. Complex network measures of brain connectivity: uses and interpretations. Neuroimage. 2010;52:1059–1069.

14. van den Heuvel MP, de Lange SC, Zalesky A, Seguin C, Yeo BTT, Schmidt R. Proportional thresholding in resting-state fMRI functional connectivity networks and consequences for patient-control connectome studies: issues and recommendations. Neuroimage. 2017;152:437–449.

15. Kimbrough A, Kallupi M, Smith LC, Simpson S, Collazo A, George O. Characterization of the brain functional architecture of psychostimulant withdrawal using single-cell whole-brain imaging. eNeuro. 2021;8:ENEURO.0208-19.2021.

16. Chen G, Chen G, Xie C, Li SJ. Negative functional connectivity and its dependence on the shortest path length of positive network in the resting-state human brain. Brain Connect. 2011;1:195–206.

17. Lancichinetti A, Fortunato S. Consensus clustering in complex networks. Sci Rep. 2012;2:336.

18. Ardinger CE, Chen Y, Kimbrough A, Grahame NJ, Lapish CC. Sex differences in neural networks recruited by frontloaded binge alcohol drinking. Addict Biol. 2024;29:e13434.

19. Guimerà R, Nunes Amaral LA. Functional cartography of complex metabolic networks. Nature. 2005;433:895–900.

20. Cohen JR, D'Esposito M. The segregation and integration of distinct brain networks and their relationship to cognition. J Neurosci. 2016;36:12083–12094.

21. Pedersen M, Omidvarnia A, Shine JM, Jackson GD, Zalesky A. Reducing the influence of intramodular connectivity in participation coefficient. Netw Neurosci. 2020;4:416–431.

22. Sporns O, Honey CJ, Kötter R. Identification and classification of hubs in brain networks. PLoS One. 2007;2:e1049.

23. van den Heuvel MP, Sporns O. Network hubs in the human brain. Trends Cogn Sci. 2013;17:683–696.

24. Humphries MD, Gurney K. Network 'small-world-ness': a quantitative method for determining canonical network equivalence. PLoS One. 2008;3:e0002051.

25. Vetere G, Kenney JW, Tran LM, Xia F, Steadman PE, Parkinson J, et al. Chemogenetic interrogation of a brain-wide fear memory network in mice. Neuron. 2017;94:363–374.e4.

26. Bloch S, Rinker JA, Smith ACW, Shiromani PJ, Wheeler DG, Azevedo R, et al. Divergent sex differences in functional brain connectivity networks in excessively drinking C57BL/6J mice. bioRxiv [Preprint]. 2021. Available from: https://doi.org/10.1101/2021.05.19.444869.

27. Lein ES, Hawrylycz MJ, Ao N, Ayres M, Bensinger A, Bernard A, et al. Genome-wide atlas of gene expression in the adult mouse brain. Nature. 2007;445:168–176.

28. Bakker R, Tiesinga P, Kötter R. The Scalable Brain Atlas: instant web-based access to public brain atlases and related content. Neuroinformatics. 2015;13:353–366.
